# Cook County Hospital, Case of Hydrophobia

**Published:** 1881-10

**Authors:** 


### Hospital Reports.

### ARTICLE X.

### COOK COUNTY HOSPITAL.

CASE OF HYDROPHOBIA. Service of DR. NORMAN BRIDGE. Reported for THE CHICAGO MEDICAL JOURNAL AND EXAMINER, by H. D. VALIN, M.D.

Peter D., a Norwegian laborer, on admission to the hospital Aug. 10, gave the following history: Father died after a surgical operation for necrosis of the tibia; mother and two sisters are living and healthy. Three children died during infancy. Patient was sick when three years old, but does not know the nature of his sickness. Had jaundice seven years ago. He has been stabbed about the face, head, back and extremities in various brawls. Has been a hard drinker, using beer to excess, and occasionally drinking whisky. Last March he was bitten on the left hand by a small dog, which was killed immediately, as a matter of course. The wound healed within three days, without a physician's care.

Monday, Aug. 8, patient worked as usual. During the afternoon he noticed that his head felt heavy, and soon began to ache, in the posterior part especially. He ate his supper, and rested well that night.

The 9th, in the morning, he ate some breakfast and drank one cup of coffee. He tried to drink another, but could not, and felt sick, with a severe headache. He lay down, with the intention to go to work at noon. At 9 A. M. a friend brought him some beer, which he attempted to drink, but could not. He then tried water, which at once made his throat feel as though he were choking. After that he could not look at water without the same feeling. Aug. 10, at night, he could not sleep well, and the 11th, in the morning, he felt worse. He said that he was smothering, and his head ached. Went out to see Dr. Fenger, and the wind made him almost wild; he had to stop often to get breath. He could not drink water or look at it without having spasms of the muscles of the throat come on. States that the place where he was bitten has caused him no pain at any time.

On admission, the 11th, he complained of pain in the head, at the base of the posterior part. A slight draught causes tremor of his whole body and a smothering feeling. Upon attempting to drink water he makes convulsive movements, and states that he feels as though he was smothering.

He had altogether, in the afternoon of the 11th, 80 grs. of chloral, in 12 and 15 grs. doses. At 7 P. M. he took 9 grs. of bisulphate of quinine. The retinæ were normal, as per ophthalmoscopic examination of Dr. Jacobson. At 10:30 P. M. he took fifteen grs. of chloral, and soon after passed his water.

At 10:45, took a drink of water by himself, a marked improvement, seemingly due to chloral. 11:30 chloral gr. xv.

Aug 11. 12:45 A. M., pulse 118. Patient cannot sleep, and takes his medicine with difficulty. All his symptoms were more marked.

2 A.M. Pulse 120.

3 A.M. A dose of chloral.

3:30. Patient was raving. Took another dose of chloral. No sleep during that night.

9:30 A.M. Chloral, gr. xv hourly. Bowels moved.

10. Pulse 128, temperature 101.8.

12 M. Half a grain of morphine hypodermically.

3:40 P.M.

℞. Potassii bromid........................ʒ j

Chloral hydrat........................gr. xv M.

one dose.

4:20. Attendant had to put on the straight jacket to keep the patient quiet.

4:25. Chloral, gr. x hypodermically.

4:53. $\frac{1}{1}$ grs. of curare was given hypodermically. The spasms came on and passed away every few minutes. Patient tried to drink water through a rubber tube four feet long, and required the goblet to be held out of sight. He did not succeed in drinking. He assumed a quarrelsome countenance, and said that he would not drink, because that would kill him, he thought. He made great efforts at expectorating, and his cough had that peculiar roughness which is also noticed in delirium tremens, and not quite unlike that of croup, though it did not resemble much the barking of a dog. Although the patient's hands and feet were tied, and he spat on his guardians, he made no effort at biting, which would naturally suggest itself in such circumstances. Although raving a part of the time, he would also occasionally speak rationally.

5:45 P.M. $\frac{1}{6}$ gr. curare hypodermically.

5:50. Chloral hydrate, gr. v., in the same manner. The patient seemed to quiet for a little while under the influence of curare, and he looked as if the disease had as yet but slightly changed his features.

6:25. Curare, gr. $\frac{1}{6}$ hypodermically. Patient soon quieted down, but still muttered.

6:35 P.M. Pulse 180. Temperature 109¼ in the rectum.

7 P.M. He breaks off the straight jacket. Pulseless at the wrist.

7:05. Aqua ammonia, ℥ss hypodermically, and also by inhalation.

7:08, temperature 109.5, pulseless.

7:10. Dead.

This case has this remarkable character, that the incubation of the disease lasted over four months, a time at which some authors believed that all dangers are passed. The duration of the disease was the average. The treatment with chloral and curare seemed beneficial.

It has been alleged that cases of death by curare have taken place, but in this we see that ten minutes before death the patient had strength enough to break off the jacket, and did not die paralyzed.

